# Supplementary material for: A Likelihood Approach for Real-Time Calibration of Stochastic Compartmental Epidemic Models
Source: PLoS Comput Biol. 2017 Jan 17;13(1):e1005257. doi: 10.1371/journal.pcbi.1005257 (PMC5240920; doi:10.1371/journal.pcbi.1005257)
Supplement: S1 File — (TAR.GZ) [file pcbi.1005257.s014.tar.gz › HSPH_Online-SI-Revision/MSS10/n1-severe/n1-severe_NEW_summary.pdf]

peak -8	R0	$1/\theta_2$	num week	inf	inflog	newly	newlylog	Reff	AttackRate
mean	30.7	52.	333.5	89.	0.3	228.9	0.4	29.5	38.8
median	30.	39.2	134.	63.1	0.3	79.4	0.3	29.8	36.3
Quantile .1	15.3	22.1	68.	41.1	0.2	42.8	0.2	14.3	24.2
Quantile .9	46.8	80.6	780.	143.7	0.5	316.	0.6	45.4	54.
36	32	36							
length	50								

peak -4	R0	$1/\theta_2$	num week	inf	inflog	newly	newlylog	Reff	AttackRate
mean	26.4	54.9	340.5	66.4	0.3	49.3	0.2	22.8	32.8
median	24.	48.1	108.5	56.	0.2	37.7	0.2	20.3	30.1
Quantile .1	11.8	17.9	53.	24.6	0.1	20.6	0.1	9.2	18.5
Quantile .9	42.8	89.7	858.	104.1	0.6	76.1	0.3	36.9	49.8
19	24	20							
length	50								

peak	R0	$1/\theta_2$	num week	inf	inflog	newly	newlylog	Reff	AttackRate
mean	22.4	59.9	425.2	83.	0.3	41.4	0.2	10.5	25.8
median	17.8	57.9	104.5	64.9	0.3	33.7	0.1	10.4	25.5
Quantile .1	7.1	13.6	50.3	20.7	0.1	18.2	0.1	2.7	12.2
Quantile .9	42.5	87.1	1454.	158.4	0.5	68.4	0.2	19.5	41.2
13	14	15							
length	50								

peak +4	R0	$1/\theta_2$	num week	inf	inflog	newly	newlylog	Reff	AttackRate
mean	10.6	42.6	469.9	61.4	0.4	42.	0.2	6.9	14.
median	10.5	43.8	143.	53.6	0.3	37.3	0.2	6.	11.1
Quantile .1	3.7	8.5	22.	20.1	0.1	19.4	0.1	1.8	3.9
Quantile .9	17.	74.5	1060.	80.1	0.6	58.8	0.4	12.1	22.9
7	10	26							
length	50								

peak +8	R0	$1/\theta_2$	num week	inf	inflog	newly	newlylog	Reff	AttackRate
mean	7.6	42.5	594.2	465.6	0.5	466.7	0.4	6.4	8.3
median	6.9	41.4	116.	65.1	0.4	48.1	0.3	5.5	6.8
Quantile .1	1.7	11.8	35.5	26.6	0.1	24.8	0.1	1.1	1.1
Quantile .9	16.	75.7	1738.	293.3	0.9	417.5	0.7	13.5	17.8
6	5	16							
length	50								
